# Mechanisms of Gravitational Influence on Weld Pool Behavior and Weld Bead Performance in Variable Polarity Plasma Arc Welding across Different Welding Position

**DOI:** 10.3390/ma16196457

**Published:** 2023-09-28

**Authors:** Jingbo Liu, Fan Jiang, Shujun Chen, Bin Xu, Guokai Zhang, Wei Cheng, Xinqiang Ma

**Affiliations:** 1 Engineering Research Center of Advanced Manufacturing Technology for Automotive Components Ministry of Education, Faculty of Materials and Manufacturing, Beijing University of Technology, Beijing 100124, China; liujbbjut@163.com (J.L.); sjchen@bjut.edu.cn (S.C.); xubin2019@bjut.edu.cn (B.X.); gkzhang@bjut.edu.cn (G.Z.); 2 Joining and Welding Research Institute, Osaka University, Osaka 567-0047, Japan; 3 Laser Institute, Qilu University of Technology (Shandong Academy of Sciences), Jinan 250353, China; 4 State Key Laboratory of Advanced Welding and Joining, Harbin Institute of Technology, Harbin 150001, China

**Keywords:** molten metal, flow behavior, three-dimensional flow, grain size, flow channel

## Abstract

This article comprehensively explores the cross-scale effects of gravity on macroscopic flow formation and weld bead formation in variable polarity plasma arc welding. Gravity-induced changes in welding direction were achieved through welding at different spatial positions. The properties of the weld bead were investigated at various spatial locations. Additionally, an elemental tracing technique was employed to study the internal flow behavior of molten metal. In the flat welding position, there is an observable trend of increasing grain size in the welded bead, accompanied by a significant expansion of the coarse grain zone. Consequently, the properties of the weld bead in the flat position are inferior to those achieved in the vertical welding position. This phenomenon can be attributed to the accumulation of molten metal at the exit side of the keyhole, resulting in temperature accumulation. Research indicates that the internal flow within the weld pool plays a critical role in causing this phenomenon. The study’s findings reveal the presence of two distinct vortex flow patterns within the weld pool: one aligned with the welding direction and the other directed towards the interior of the weld pool. Particularly noteworthy is the substantial expansion of the flow channel area in the flat welding position, which significantly amplifies the impact of internal flow. This enhanced flow intensity inevitably leads to the increased buildup of molten metal at the keyhole exit side. These studies lay the groundwork for achieving high-quality and controllable spatial-position welding.

## 1. Introduction

Variable polarity plasma arc (VPPA) welding gained widespread application in the field of aluminum alloy structural connections due to its cathode cleaning effect and concentrated arc energy characteristics [1,2,3,4,5,6]. Compared to traditional arc welding techniques, VPPA welding, with its unique keyhole welding mode, effectively eliminates porosity, resulting in superior welding quality and properties of welding bead [7]. Consequently, VPPA welding exhibits special adaptability in areas requiring high-quality welds, such as aerospace and large ship manufacturing [8]. However, the intricate welding mode associated with keyhole also constrains the development of VPPA welding technology [9]. The presence of keyhole makes the flow of molten metal within the weld pool more complex, which could lead to weld pool collapse and welding instability [10]. In practical engineering applications, as workpiece volumes increase, operations such as flipping or moving become challenging. Therefore, repositioning the welding torch for flat welding position becomes unavoidable. Many scholars propose that vertical welding is the optimal position for VPPA welding, as it allows molten metal to flow and solidify more effectively within the weld pool, resulting in high-quality welds [11,12]. However, during flat welding, the change in gravity direction disrupts force equilibrium, potentially increasing welding process instability and affecting the properties of welding bead [13]. Hence, investigating the influence of gravity on the molten metal in the flat VPPA welding becomes crucial.

Several scholars conducted research on the properties of VPPA-welded beads [14,15]. Cai et al. pointed out that the properties of VPPA-welded beads surpass those of traditional arc-welded beads [16]. Yan et al. investigated the influence of different torch angles on bead performance, highlighting that adjusting the flow behavior of molten metal and enhancing the performance of VPPA-welded beads in the transverse welding configuration can be achieved by altering the torch angle [17]. However, transverse welding is more adjustable due to evident gravitational differences, which contrasts with horizontal welding. In the case of horizontal welding, as gravity acts on the pool’s exit side and is challenging to optimize through changes in welding parameters or other conditions, exploring the flow behavior and properties of VPPA welding in the horizontal position holds significant importance for the successful industrial application of this advanced technique. During the welding process, the flow and solidification of molten metal significantly influence the properties of welded beads, thereby impacting the properties of welding beads [18,19,20].

Numerous scholars conducted research on VPPA welding in different welding positions. Maintaining the flow behavior of molten metal is considered a key factor in ensuring welding stability [21,22], making the study of molten metal flow within the weld pool crucial [23,24,25]. Wu et al. established a multi-factor-coupled model through simulation to investigate the influence of different driving forces on the weld pool flow behavior during keyhole welding [26]. They pointed out that arc shear force and arc pressure are the main driving forces that induce directed flow of molten metal within the weld pool. Liu et al. utilized oxidized wires in the welding process to release oxide particles and track their flow behavior [27]. They confirmed that in vertical welding, gravity assists in better guiding the flow of molten metal towards solidification at the rear of the weld pool, with flow occurring from the keyhole front side to the rear side. Morisada et al. used metallic tungsten as a tracer particle to capture the flow behavior of the weld pool during flat welding [28]. The material flow was obtained by three-dimensional visualization through X-ray radiography. Ahn et al. used metal tungsten particles and an X-ray system to capture internal flow patterns of the weld pool, identifying arc shear force as the primary driving force during welding [29]. However, due to the significantly higher density of metal tungsten particles compared to molten metal, this method may not accurately reflect internal flow dynamics. In order to obtain the flow trajectory of the metallic material, Mugada et al. characterized the flow behavior of the base material by incorporating copper metal into it to trace the flow trajectory of copper metal [30]. However, this approach is only applicable to solid-state joining processes, as introducing copper into the molten pool during arc welding would compromise its stability. Yan et al. employed elemental tracing, adding Cu elements to form Al-Cu compounds during welding, and tracked their flow behavior within the weld pool [31]. Al-Cu compounds naturally exist in both the pool and weld, providing a more precise representation of internal flow dynamics. Nevertheless, the mentioned studies mainly focused on macroscopic pool flow, lacking research on microstructural performance, particularly the cross-scale impact of flow-induced microstructural changes in properties of welding bead.

This study focuses on examining the variations in the mechanical properties of welded beads at different welding positions, and aims to analyze and discuss the underlying reasons for these differences based on flow behavior. Specifically, the investigation delves into the gravitational influence on the internal flow dynamics and properties of aluminum alloy VPPA welding. To capture the flow patterns within the weld pool, the elemental tracer method was employed. Leveraging three-dimensional reconstruction techniques, the study unveiled the flow patterns within the spatial domain of the weld pool. Furthermore, the study explored the mechanism through which gravity influences the flow of molten metal during the variable polarity plasma arc welding process. The outcomes of this research provide valuable insights for enhancing the horizontal welding process of aluminum alloy VPPA welding.

## 2. Materials and Methods

### 2.1. Common Experimental Setup and Welding Conditions

**Figure 1:**
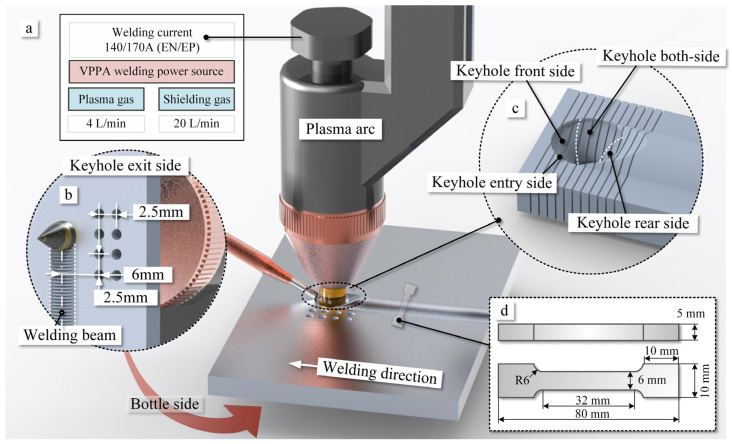
Schematic diagram of the experimental VPPA welding system. (**a**) Schematic diagram of the VPPA welding system. (**b**) Distribution of temperature measurement points on both sides of the weld pool. (**c**) Schematic diagram of the keyhole wall. (**d**) Schematic diagram of the sampling position.

The experimental design for VPPA welding is illustrated in Figure 1. The welding system parameters include welding current, plasma gas, and shielding gas, set at 140/170 A (EN/EP), 4 L/min, and 20 L/min, respectively, as shown in Figure 1a. Both the plasma gas and shielding gas used are argon. The welding torch traverse speed and filler wire feed speed are 15 cm/min and 100 cm/min, respectively. The experimental setup is positioned in two spatial orientations: flat and vertical. In the flat position, the plate is parallel to the ground, the torch is perpendicular to the plate, and gravity is perpendicular to the direction of the weld, as shown in Figure 1. In the vertical position, the torch maintains its position relative to the base metal, while the plate surface is perpendicular to the ground, and gravity is opposing the direction of the weld. Furthermore, the side forming the weld seam corresponds to the keyhole rear side, with the opposite side being the front wall of the pool. The keyhole entry side, which comes into direct contact with the arc, represents the top side, and the keyhole exit corresponds to the bottom of the keyhole.

The base material chosen is a 5052-aluminum alloy with dimensions of 5 mm × 200 mm × 200 mm. In order to minimize experimental errors during both the in-process and post-welding stages of wall welding, filler wire ER5183 with the same chemical composition as the base material was used for the structural performance experiments. The detailed chemical composition of the selected material is provided in Table 1.

### 2.2. Mechanical Properties of Welding Bead

To assess the strength of weld beads and understand the influence of internal flow within the weld pool on mechanical properties, weld bead specimens were cut across the welding direction, as depicted in Figure 1d. Following corrosion, the cross-sections of the weld beads were captured using laser confocal microscopy, allowing measurement of grain size and distribution. For testing mechanical properties, specimens with specific dimensions were employed, as shown in Figure 1d. The fracture surfaces of tension tests were obtained using scanning electron microscopy Quanta 650 FEG (Thermo Fisher Scientific, Waltham, MA, USA), allowing measurement of elongation and tensile strength of the welded beads.

### 2.3. Measurement of Molten Metal Flow in the Weld Pool

**Figure 2:**
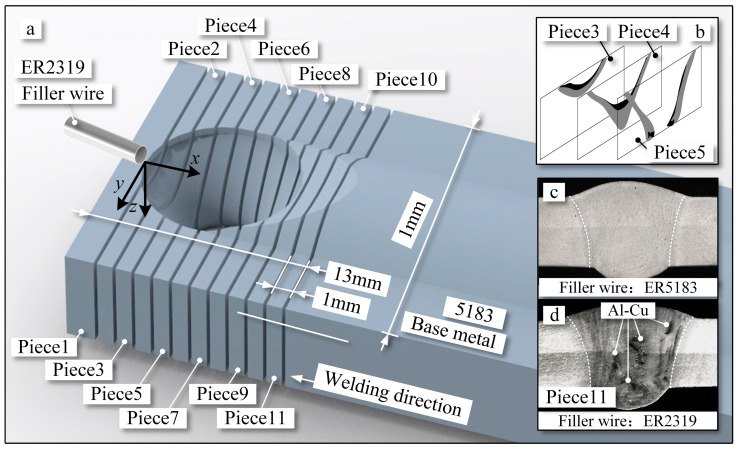
Schematic illustration of keyhole and weld pool pieces. (**a**) Schematic illustration of weld pool pieces. (**b**) Schematic diagram of slice reconstruction. (**c**) Results of cross-sectional corrosion of the weld seam with ER5183 filler wire. (**d**) Results of cross-sectional corrosion of weld seam with ER2319 filler wire.

To capture the flow behavior within the weld pool, the elemental tracing method and axial tomography method were applied. The elemental tracing method involved the introduction of compounds into the weld pool capable of characterizing molten metal flow. The axial tomography method facilitated the three-dimensional reconstruction of these characterized elemental compounds, as depicted in Figure 2. Similar to the attributes of the welding base material, filler wire ER2319 was utilized, with the unique inclusion of the element Cu, as depicted in Table 1. Additionally, the chosen Cu element can readily form compounds with Al. These selected compounds were etched using Keller reagent. Among the composed compounds, the grain boundaries of the Al-Cu compound have the highest energy, resulting in their preferential corrosion, as shown in Figure 2d, when the etching process is controlled for a fixed duration. By doing so, the distribution of the Al-Cu compound at different welding pool sections could be preserved, facilitating the tracking of internal flow within the weld pool. Furthermore, when ER5183 filler wire, similar in composition to the base material, was added, it was used for assessing structural and mechanical properties, as depicted in Figure 2c. Due to the absence of Cu in ER5183, there is no occurrence of the black region corresponding to Al-Cu compounds.

Through laser confocal microscopy, a sequential imaging of cross-sectional slices of solidified weld pool keyholes was performed, enabling the axial tomography of oxide compounds, as shown in Figure 2b. Rapid shutdown of the welding power source was achieved by cutting off the supply of arc and shielding gas from the welding machine. This method efficiently induced the instantaneous oxidation and solidification of the molten metal within the weld pool, preserving the state of the weld pool keyhole during welding. For the observation of Al-Cu compound distribution, a total of 11 cross-sections were cut from the front to back of the keyhole, as illustrated in Figure 2a. The dimensions of the keyhole are 12 mm in length and 10 mm in width. Each cut piece measures 5 mm × 15 mm × 1 mm, as shown in Figure 2a. Employing this approach, the distribution of the Al-Cu compound in the current piece can be obtained. Each etched piece was photographed using laser confocal microscopy, as shown in Figure 2d. Based on the acquired distribution of Al-Cu compounds, a three-dimensional reconstruction yields the compound distribution within the weld pool keyhole. Furthermore, the origin of the coordinate system is set at the edge of the weld pool, where the filler wire is introduced into the pool. The x-direction represents the opposite direction of welding, the y-direction points toward the side wall of the pool, and the z-direction indicates the depth of the weld pool.

### 2.4. Measurement of Temperature Distribution in the Weld Pool

Thermocouples were positioned on one side of the weld pool because in both flat and vertical positions, the molten metal flow within it is symmetrical, as shown in Figure 1. Temperatures were measured at distances of 6 mm and 8.5 mm from the weld seam edge. Multiple thermocouple measurement points and repeated experiments were employed to minimize the impact of errors on the experimental results.

## 3. Results

### 3.1. Properties of Welding Bead

**Figure 3:**
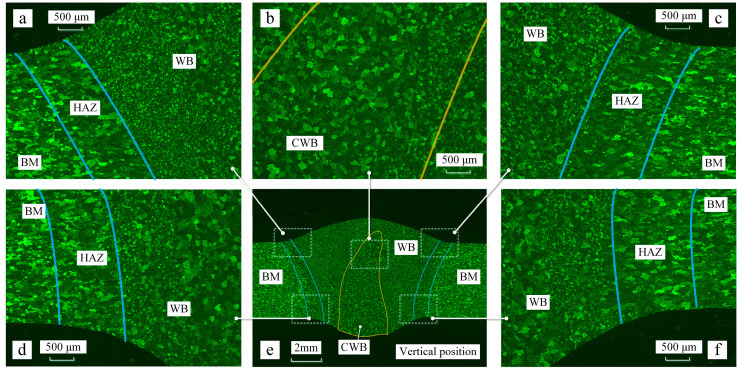
Properties of the welding bead in the vertical position. (**a**) Properties of the part of the welding bead on the upper left side. (**b**) Properties of the part of the welding bead on the middle side. (**c**) Properties of the part of the welding bead on the upper right side. (**d**) Properties of the part of the welding bead on the lower left side. (**e**) Properties of the welding bead. (**f**) Properties of the part of the welding bead on the lower right side.

Figure 3 illustrates the properties of the welded bead during vertical welding. Figure 3a–d,f depicts the properties at different positions of the welding bead during the vertical welding position. The top side corresponds to the side away from gravity. There are variations in grain distribution across different zones. The base metal (BM) displayed compressed grains oriented in line with the rolling direction, indicative of its manufacturing process. In both the heat-affected zone (HAZ) and weld bead (WB), a characteristic as-cast solidification structure was evident, characterized by the presence of equiaxed grains. A parallel observation was made on the top side, except for the HAZ, as shown in Figure 3a,c,d,f. In the weld bead center (CWB), the presence of a coarser-grained zone, such as the columnar-to-equiaxed transition, was noted. Its area was measured to be 26.6 mm². During vertical welding, the CWB exhibits a droplet-shaped pattern, with a wider area near the weld root and a narrower area near the weld crown. For the vertical position, the crown height and root penetration were measured to be 1.8 mm and 1.1 mm, respectively.

**Figure 4:**
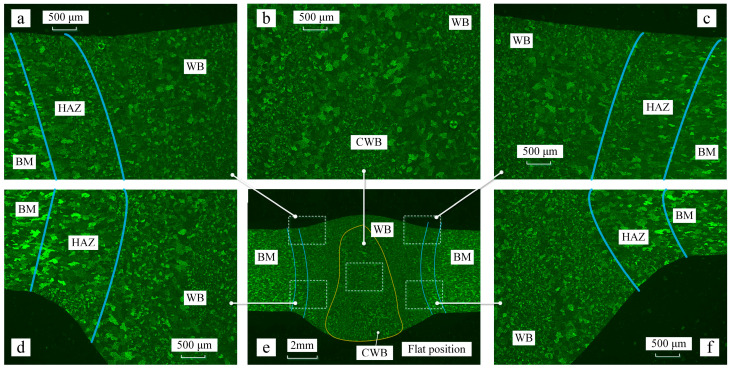
Properties of the welding bead in the flat position. (**a**) Properties of the part of the welding bead on the upper left side. (**b**) Properties of the part of the welding bead on the middle side. (**c**) Properties of the part of the welding bead on the upper right side. (**d**) Properties of the part of the welding bead on the lower left side. (**e**) Properties of the welding bead. (**f**) Properties of the part of the welding bead on the lower right side.

The grain distribution pattern during the flat welding position is similar to that during the vertical welding position, as shown in Figure 3e and Figure 4e. Properties at different positions of the welding bead during the vertical welding position are depicted in Figure 4a–d,f. The coarse-grained zone in flat welding is also concentrated in the center of the weld, with an area of 46.2 mm^2^. During flat welding, the CWJ shows a square distribution, evenly spread across the center of the weld pool. However, in the flat welding position, the size of the area near the weld root is larger than that in vertical welding. The central coarse-grained zone in flat welding is larger than that in vertical welding. For the flat position, the crown height and root penetration were measured to be 0.7 mm and 2.3 mm, respectively.

**Figure 5:**
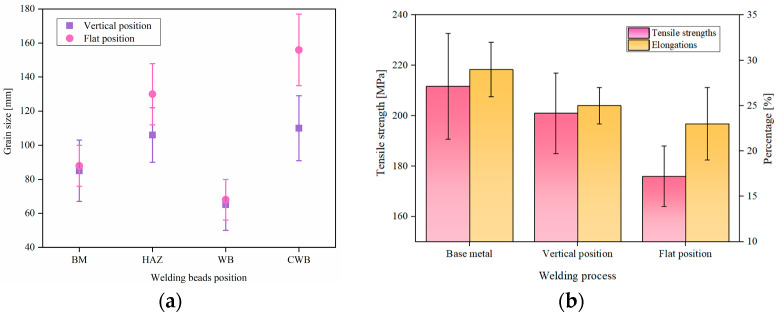
(**a**) Grain size of the welding bead. (**b**) The tensile strength and elongation of welds.

The grain size was determined using the standard linear intercept method, which is the primary technique for determining grain size. The grain sizes on the lower side of the welded bead were almost the same. However, the grain sizes varied with the welding position within the range of error. The grain size of the base material was around 88 μm, as showed in Figure 5a. For vertical welding position, the sizes of the HAZ, WB, and CWB were measured to be 106 μm, 66 μm, and 126 μm, respectively. In contrast, for flat welding, these sizes were 110 μm, 70 μm, and 156 μm, respectively. The grain sizes increased by 3.7%, 6%, and 23.8% for the HAZ, WB, and CWB, respectively, during flat welding. Particularly, in the vertical welding position, the average grain size in the weld center was larger than the average grain size in the weld bead by 20 μm, while in flat welding, this difference increased to 46 μm. The variation in grain size at the center of the welded bead was smaller compared to the flat welding position.

Furthermore, the mechanical properties of the welded bead were measured, as shown in Figure 5b. Five samples were taken for each welding position. For vertical welding, the tensile strength of the weld bead was 202 MPa, and for flat welding, it was 176 MPa. Both values were lower than the tensile strength of the base material (213 MPa). The tensile strength during flat welding was significantly lower than that during vertical welding. The elongation of the base material was 28%, while for vertical and flat welding, the elongation rates were 25% and 23%, respectively. The elongation rates of both welding positions were slightly lower than that of the base material, and the elongation rate in flat welding was still lower than that in the vertical welding position.

**Figure 6:**
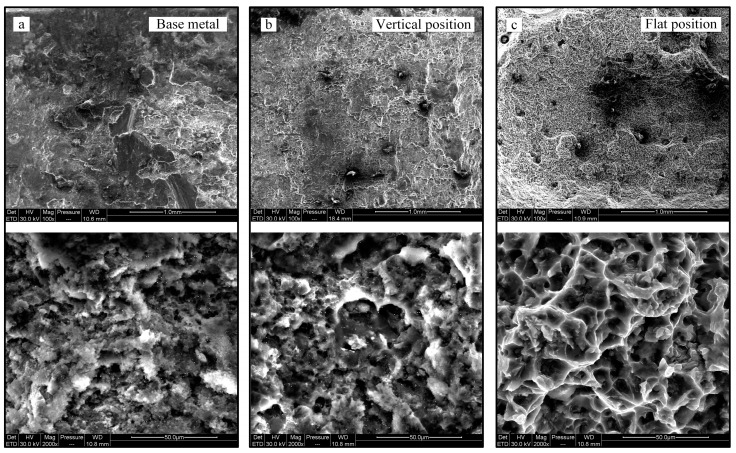
SEM fractography of the fractured surfaces. (**a**) Base metal. (**b**) Vertical position. (**c**) Flat position.

The SEM images of the tensile fracture surfaces at different welding positions are shown in Figure 6. The tensile result of the base material serves as a reference. The fracture surfaces exhibit extensive tearing, dimples, and clear indications of fracture, as depicted in Figure 6a. The reference outcome from the BM displays a fracture surface exhibiting numerous tears and dimples, indicative of a quasi-cleavage fracture. In both vertical position and BM, equiaxial dimples manifest on the fracture surface of the welded bead, depicted in Figure 6a,b. Conversely, in the case of the flat welding position, torn dimples become apparent. All these instances exhibit ductile fracture characteristics under tensile testing conditions. Notably, within the flat welding position, specimens fractured at the HAZ of the welds, whereas the BM and vertical position experienced random fractures.

### 3.2. Internal Flow Behavior within the Weld Pool

**Figure 7:**
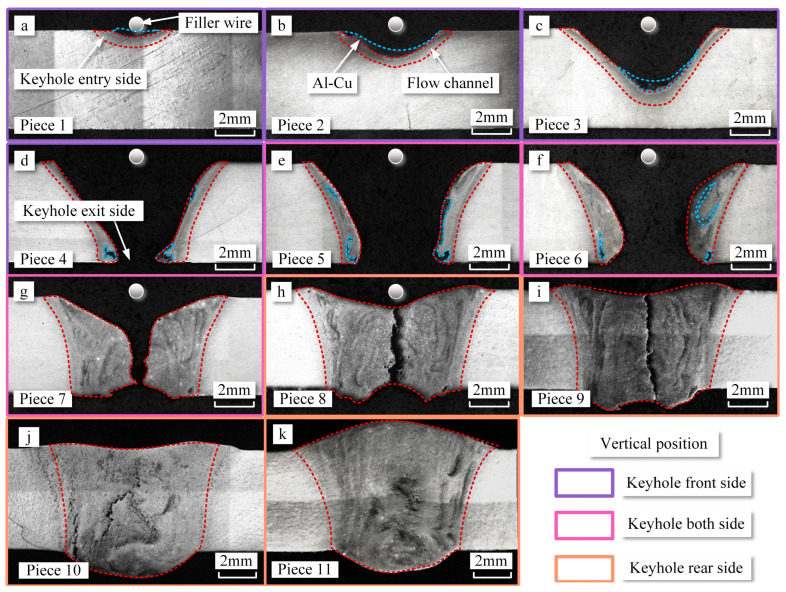
The internal flow behavior of molten metal in the vertical position. (**a**) Piece 1. (**b**) Piece 2. (**c**) Piece 3. (**d**) Piece 4. (**e**) Piece 5. (**f**) Piece 6. (**g**) Piece 7. (**h**) Piece 8. (**i**) Piece 9. (**j**) Piece 10. (**k**) Piece 11.

During vertical welding position, the distribution of the Al-Cu compound within the weld pool keyhole is depicted in Figure 7. Pieces 1 to 11 represent cross-sections along the welding direction of the keyhole. Pieces 1 to 4 correspond to the keyhole front side of the weld pool, as shown in Figure 7a–d. In these figures, the red zones denote the flow channels, while the blue areas indicate the Al-Cu compound. In Figure 7a, the filler wire is introduced into the weld pool, and upon its melting, it becomes mixed with the molten metal generated from the base material. As the welding progresses, molten metal is continually conveyed into the weld pool. The metal flows along the edges of the keyhole, moving from the keyhole entry side to the keyhole exit side, as illustrated in Figure 7b–d.

While the flow on either side of the weld is not perfectly symmetric in actual welding, the overall flow channels are quite consistent along both sides of the weld. To facilitate the comparison of the changes in flow channels from the front wall to the rear wall of the weld pool, the actual area is calculated as half of the flow channel area. This study focuses on both a vertical and flat position, making the flow of molten metal relatively symmetric on both sides of the weld pool. The flow channel areas for Piece 1 to Piece 4 are 3.78 mm^2^, 4.31 mm^2^, 4.84 mm^2^, and 5.46 mm^2^, respectively. The flow channel area at the front wall increases by 44%.

**Figure 8:**
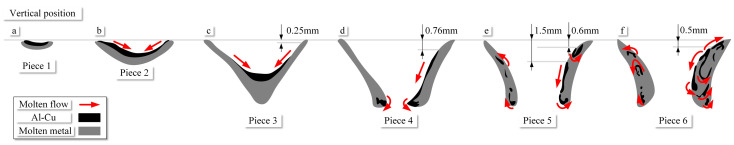
Schematic illustration of molten metal flow in the vertical position (**a**) Piece 1. (**b**) Piece 2. (**c**) Piece 3. (**d**) Piece 4. (**e**) Piece 5. (**f**) Piece 6.

From Pieces 1 to 6, the welding direction is reversed, which corresponds to the primary direction of molten metal flow. The observation of metal flow within the weld pool keyhole is based on differences in the distribution of Al-Cu compounds. After extraction, a schematic representation of this process is illustrated in Figure 8. The accumulation of Al-Cu compounds in the black zone occurs on the surface of the weld pool, consistently remaining near the bottom of the current piece, as shown in Figure 8a–c. Tracing elements on the same side exhibit continuity. As a result, the depths of the weld pool front wall in Pieces 3 and 4 from the keyhole entry side are 0.25 mm and 0.76 mm, respectively, as depicted in Figure 4c,d. Within the flow channel, Al-Cu compounds exhibit an alternating arrangement, forming a regularly patterned vortex street phenomenon.

Pieces 5 to 7 represent sections of the keyhole on both sides, as depicted in Figure 7e–g. Moving from the keyhole front side to both sides, the area of the flow channel gradually increases. Al-Cu compound near the keyhole front side flow along the wall surface. In the weld pool sidewall, some Al-Cu compounds exhibit flow from the wall surface into the interior of the weld pool. Al-Cu compounds flow along the pool wall to the keyhole exit side and generate vortices within the pool interior at the bottom, as shown in Figure 8d–f. Furthermore, with the increase in the flow channel area, internal flow also occurs within the pool wall, as depicted in Figure 8e,f. Al-Cu compounds contribute to the inward flow. In Piece 7, near the keyhole rear side, the diffusion of Al-Cu compounds extends throughout the entire interior surface of the pool wall. The flow channel areas for Pieces 5 to 7 are 5.78 mm^2^, 7.36 mm^2^, and 13.74 mm^2^, respectively. The largest flow channel in the weld pool sidewall is 137.77% larger than that in the front wall. 

Particularly, due to the method of arc disruption used to capture the keyhole’s morphology during welding, the weld pool keyhole exhibits cracks when suddenly losing heat input, as illustrated in Figure 7h. An interesting phenomenon was observed in the weld pool sidewall: the tracing elements at a depth of 0.6 mm from the wall’s surface exhibit bifurcation. Some tracing elements create vortices flowing inward along the wall, while another group flows toward the keyhole exits side, generating a second vortex.

**Figure 9:**
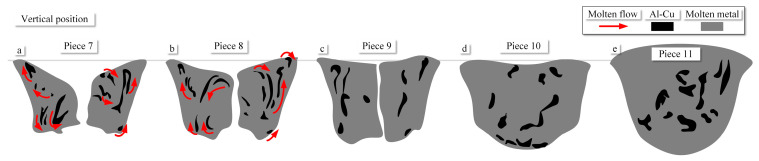
Schematic illustration of molten metal flow in the vertical position. (**a**) Piece 7. (**b**) Piece 8. (**c**) Piece 9. (**d**) Piece 10. (**e**) Piece 11.

Pieces 8 to 11 correspond to sections of the keyhole rear side, as illustrated in Figure 7h–k. The flow channel areas for Pieces 8 to 11 are 19.44 mm^2^, 23.13 mm^2^, 24.80 mm^2^, and 28.26 mm^2^, respectively. The largest flow channel in the keyhole rear side is 106% larger than that in the keyhole on both sides. However, the flow channel area on the keyhole rear side only increases by 45%. From Piece 8 to Piece 11, as the solidification zone shifts to the keyhole rear side, the weld bead’s crown and root form gradually. The specific flow patterns within the weld pool are challenging to discern. However, their distribution patterns can be easily obtained. Al-Cu compounds are distributed across the entire keyhole rear side. After the formation of the weld bead, when the molten metal fully solidified, Al-Cu compounds are predominantly distributed throughout the cross-section, as shown in Figure 9. It can be observed that within the keyhole rear side, Al-Cu compounds are uniformly distributed at its center. However, as solidification progresses, Al-Cu compounds primarily distribute across the entire surface of the keyhole rear side.

**Figure 10:**
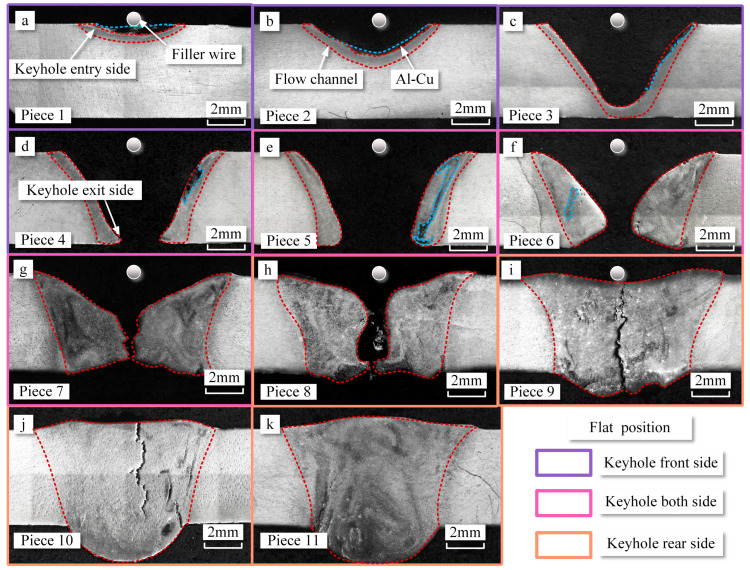
The internal flow behavior of molten metal in the flat position. (**a**) Piece 1. (**b**) Piece 2. (**c**) Piece 3. (**d**) Piece 4. (**e**) Piece 5. (**f**) Piece 6. (**g**) Piece 7. (**h**) Piece 8. (**i**) Piece 9. (**j**) Piece 10. (**k**) Piece 11.

During the flat position, the distribution of the Al-Cu compound in the weld pool cavity is shown in Figure 10. In these figures, the red zones denote the flow channels, while the blue areas indicate the Al-Cu compound. Similar to the vertical welding position, Pieces 1 to 4 represent the keyhole front side, as shown in Figure 10a–d. The flow channel areas for Piece 1 to Piece 4 are 3.75 mm^2^, 4.54 mm^2^, 4.99 mm^2^, and 5.2 mm^2^, respectively, indicating an increase in area by 38%. The difference in flow channel areas for the same slices compared to vertical welding is only 8%. Al-Cu compound accumulates on the surface of the weld pool, which is consistent with the vertical welding position.

**Figure 11:**
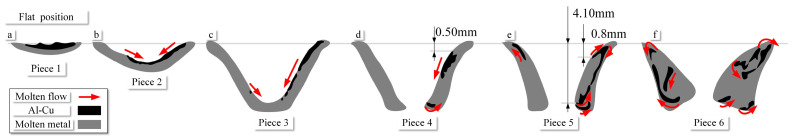
Schematic illustration of molten metal flow in the flat position. (**a**) Piece 1. (**b**) Piece 2. (**c**) Piece 3. (**d**) Piece 4. (**e**) Piece 5. (**f**) Piece 6.

The distribution schematic of the Al-Cu compound in the weld pool cavity during flat welding is shown in Figure 11. The Al-Cu compound adheres to the weld pool surface and flows along the front wall of the pool, moving from the welding entry side to the keyhole exit side. Flow towards the interior of the pool occurs at the junction between the front wall and the side wall, as shown in Figure 11d,e. Additionally, inward vortices appear at positions towards the exit side of the pool. Along the side wall of the pool, the flow channel areas for Pieces 5 to 7 are 6.02 mm^2^, 9.5 mm^2^, 16.12 mm^2^, and 16.13 mm^2^, respectively, exhibiting a 167% increase in flow channel area from the keyhole front side to the both-side wall. Correspondingly, these areas in vertical welding at the respective positions increased by 16.2%, 64.3%, 119%, and 173.39%, indicating a significant increase in flow channel size during flat welding at the same piece positions.

Near the exit side of the keyhole exit side, vortices also emerge, causing the inward flow of Al-Cu towards the interior of the pool. Simultaneously, the sudden increase in the flow channel on the side wall causes earlier diffusion of the Al-Cu compound. During flat position, a separation point on the side wall of the pool is discovered at a depth of 0.8 mm. The first internal vortex appears here, followed by a second vortex towards the exit side, as depicted in Figure 11e.

**Figure 12:**
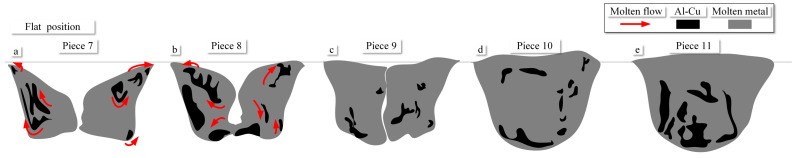
Schematic illustration of molten metal flow in the flat position. (**a**) Piece 7. (**b**) Piece 8. (**c**) Piece 9. (**d**) Piece 10. (**e**) Piece 11.

In the position of flat welding, for the keyhole rear side, the flow channel areas for Pieces 8 to 11 are 17.4 mm^2^, 22.94 mm^2^, 24.12 mm^2^, and 28.1 mm^2^, respectively, as depicted in Figure 12e. The changes in flow channels at the rear wall are relatively small. These areas, corresponding to the vertical welding’s respective positions, were reduced by 10%, 0.8%, 2.7%, and 0.5%. The variations in flow channels at the keyhole rear side are minor. After the formation of the weld, the Al-Cu compound is primarily distributed near the exit side of the cross-section, showing significant differences compared to vertical welding.

### 3.3. Temperature Distributions on the Weld Pool Surfaces

**Figure 13:**
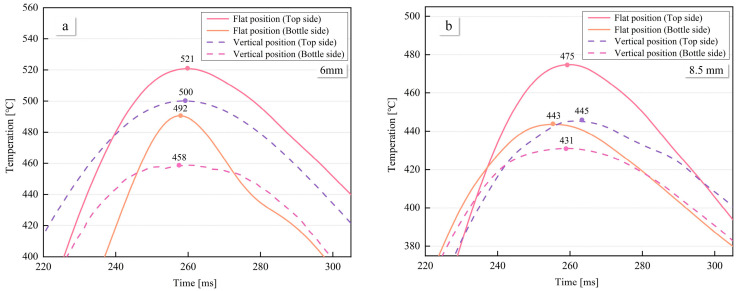
The average temperature distribution on the top and bottle side of the heat-affected zone of the weld pool. (**a**) The average temperature distribution of the weld pool at a distance to weld seam canter of 6 mm. (**b**) The average temperature distribution of the weld pool at a distance to weld seam canter of 8.5 mm.

Temperature variation in HAZ of the weld seam at different welding positions is illustrated in Figure 13. The temperature change at 6 mm from the weld seam is depicted in Figure 13a, where the maximum temperatures for the top and bottle side of the flat position are 521 °C and 492 °C, respectively. The maximum temperature difference between the top and bottle side of the flat position is 29 °C. In contrast, for the vertical position, the maximum temperatures for the top and bottle side of the weld pool are 500 °C and 458 °C, respectively, resulting in a maximum temperature difference of 42 °C between the top and bottle sides. Consequently, the temperature difference between the top and bottle sides of the weld is smaller in the flat position compared to vertical welding. Notably, both for vertical and flat position welding, the temperature on the top side of the weld is higher than that on the bottle side. Moreover, the temperatures on both sides of the flat position are higher than the corresponding sides of the vertical position.

The temperature change at 8.5 mm from the weld seam is shown in Figure 13b, where the maximum temperatures for the top and bottle side of the flat position are 466 °C and 443 °C, respectively, resulting in a temperature difference of 23 °C. For the vertical position, the temperature difference between the front and back sides is 27 °C. The lower temperatures and temperature differences at 8.5 mm compared to 6 mm are attributed to the increased distance from the weld pool.

## 4. Discussion

**Figure 14:**
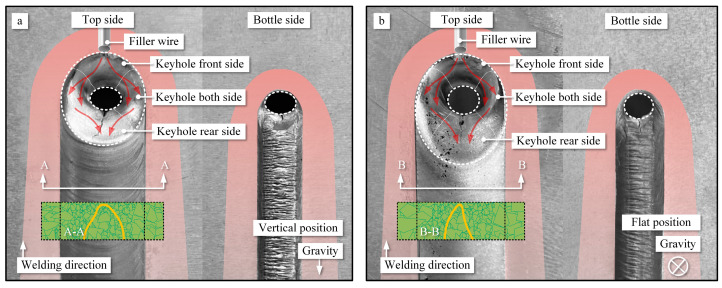
Schematic diagrams of weld pool flow patterns, temperature distributions and grain distributions at different welding positions. (**a**) Vertical position. (**b**) Flat position.

In VPPA welding, the flow patterns of the weld pool, temperature distribution, and grain distribution are illustrated in Figure 14. The distribution of grains on the left and right sides of the weld pool is similar during both vertical and flat welding, as shown in Figure 14. This similarity arises from the uniform distribution of the arc’s effect on both sides of the keyhole. However, variations in temperature differences along the keyhole front side and rear side in different welding positions lead to differences in grain growth.

For the free growth model, the grain size is described by the following formula:

(1) $$d=\frac{4\delta }{{∆T}_{f}{∆S}_{f}}$$

where d, ${∆T}_{f}$, δ, and ${∆S}_{f}$ represent the grain size, undercooling, interfacial energy, and entropy of fusion, respectively. It means that at a clear ${∆S}_{f}$, which is inversely proportional to grain size, the grains begin to develop freely from these. This model claims that grain growth is solely temperature-dependent and independent of time.

According to this model, it indicates that grains begin to grow freely under a specific degree of undercooling, and their size is inversely proportional to the diameter. Based on this model, grain growth is independent of time and solely dependent on temperature. In the flat position, due to the overall higher temperature of the weld pool compared to vertical welding, and the smaller temperature difference between the top and bottle of the weld pool, the cooling rate is slower. This leads to larger grain sizes in flat welding. However, the excessively high temperature distribution results in slower grain production, further contributing to reduced mechanical properties of the welded bead. Consequently, the tensile elongation and tensile strength of the welded bead in flat welding are lower than those in vertical welding. 

In the vertical welding position, the zone of coarse grain near the keyhole exit side is smaller, whereas in the flat welding position, the area of coarse grains is larger and concentrated in the middle. This phenomenon can be attributed to the temperature differential inherent in vertical welding processes, wherein the temperature gradient between the upper and lower regions of the molten metal is pronounced, resulting in accelerated solidification at the exit side of the keyhole. Variations in temperature distribution across distinct welding positions can be attributed to disparities in the dynamics of the weld pool, which, in turn, are influenced by gravitational changes. These gravitational alterations consequently impact grain distribution.

**Figure 15:**
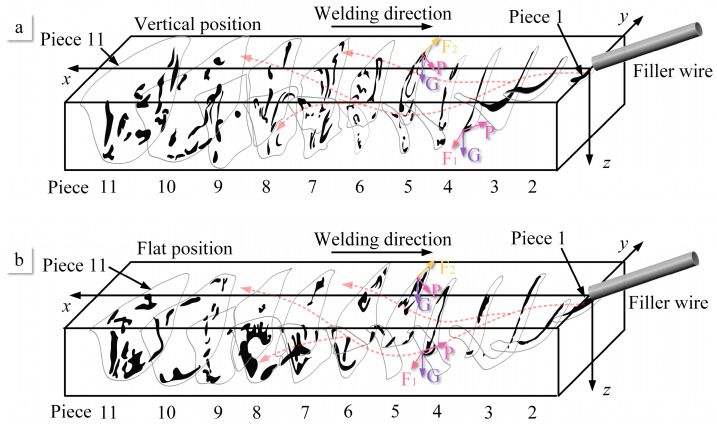
Three-dimension reconstruction of the weld pool pieces. (**a**) Vertical position. (**b**) Flat position.

The flow of molten metal is reconstructed through the process of sectioning and three-dimensional reconstruction of the weld pool, as illustrated in Figure 15. Along the x-direction, which corresponds to the opposite direction of welding, spanning from the region proximate to the welding wire to the far side, encompassing weld pool pieces 1 through 12, the reconstructed sections offer a comprehensive insight into the internal dynamics of the molten metal within the keyhole’s leading edge, side walls, as well as the accumulation at the rear side of the keyhole. The motion of the molten metal is driven by a diverse array of forces, which encompass shear forces, gravity, arc pressure, Lorentz forces, Marangoni forces, and others, as extensively discussed in reference [32]. Among these, the dominant forces driving the flow in the weld pool are the arc shear forces, gravity, and arc pressure [33]. Hence, this study primarily delves into the discussion of the three aforementioned key driving forces and their effects on various flow behaviors.

The arc shear force is a result of the directed flow of the plasma arc, causing the molten metal to flow. Due to the distinctive shape of the weld pool with a wide keyhole entry side and a narrow keyhole exit side, there exists a countercurrent flow of the plasma arc within the pool. This countercurrent flow generates two opposite deflections on the weld pool walls: one directed towards the entrance side of the pool (F1) and the other towards the exit side of the pool (F2), as depicted in Figure 15. According to the simulation results by Wu et al., it was found that the arc shear force directed towards the keyhole exit side is smaller than that directed towards the keyhole entry side [31]. Gravity (G) acts vertically downward towards the horizontal ground and is considered the second most influential factor affecting the flow in the weld pool. Arc pressure (p) points from the liquid surface towards the center of the pool, as demonstrated by Wu et al.’s research [33]. However, due to the presence of the conical keyhole, the direction of arc pressure at different positions on the weld pool’s wall varies. Specifically, this results in opposing directions of arc pressure at the keyhole entry side and the keyhole exit side.

**Figure 16:**
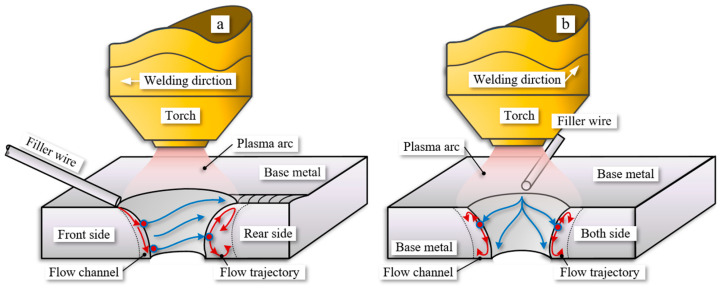
Schematic of the molten metal flow inside the VPPA weld pool. (**a**) Vertical position. (**b**) Flat position.

The primary flow pattern of the molten metal within the weld pool involves flowing from the keyhole front side to the keyhole both side and then towards the keyhole rear side. At the front wall of the weld pool, the narrowness of the flow channels confines the movement of the molten metal due to applied forces, as illustrated in Figure 16a. The blue arrows show the flow behavior at the surface of the molten pool and the red arrows show the flow behavior inside the molten pool. On the upper side of the weld pool, the effects of gravity and arc pressure on the front wall are more significant than the arc shear force F1. As a result, the molten metal flows from the keyhole front side to the keyhole exit side. Simultaneously, near the keyhole both-side wall, the direction of the gravitational and arc shear force components on the lower side of the weld pool aligns, thus causing the molten metal to flow towards the exit side of the pool. Due to the similarity in the flow channels on the front side of the keyhole, the differences in flow patterns between the vertical and flat welding positions are relatively small. Gravity has a minor impact on the flow of molten metal on the front wall of the weld pool.

The flow patterns of molten metal on the keyhole both-side wall can be divided into two types: the first involves the flow from the sidewalls to the rear wall of the pool, while the second pertains to the occurrence of internal vortex flow. Vortex flows are observed at two positions within the weld pool: one near the entrance side and the other near the exit side of the pool, as shown in Figure 16b. The vortex flow near the keyhole entry side is a result of the combined effects of gravitational force and arc pressure, causing a portion of the molten metal along the pool wall to undergo inward convection. On the other hand, the vortex flow near the exit side is attributed to the limited liquid wall area, leading to an accumulation of molten metal in this zone. The forces at this location, including gravity, arc shear force, and arc pressure, all direct towards the interior of the pool, thus enhancing the prominence of the vortex flow. 

In the flat position, the influence of gravity drives the molten metal to flow more towards the keyhole exit side than towards the keyhole rear side, as indicated in Piece 6 of Figure 15. As a result, the disparity in the flow channel area within the weld pool is considerably more conspicuous in the flat welding position when juxtaposed with the vertical position. Furthermore, this phenomenon precipitates the earlier onset of internal convection within the weld pool during flat welding operations. In the context of VPPA welding, the existence of internal vortices instigated by the prevailing driving forces amplifies the active agitation of molten metal within the weld pool. This phenomenon, in turn, plays a pivotal role in augmenting the defect mitigation and enhancing the microstructural performance of VPPA welding when contrasted with alternative welding methodologies.

Due to the further expansion of the flow channels at the keyhole rear side, the internal flow within the weld pool becomes challenging to track. However, the internal vortices within it can still be discerned. Based on my previous research, two opposing flow patterns exist for the molten metal at the rear side: one flowing towards the exit side of the pool and the other flowing towards the entry side [27]. Therefore, in conjunction with the results of this experiment, it can be observed that there are two types of internal vortices within the rear wall of the weld pool. One type flow towards the keyhole entry of the pool and generates an internal vortex that flows inward, while the other type flows towards the keyhole exit side and forms an inward vortex near the keyhole exit side, as shown in Figure 16a. This flow behavior aligns with the simulation results by Wu et al. [26]. The combined effects of arc shear force, arc pressure, and gravity lead to a clockwise internal vortex near the keyhole entry side. Conversely, due to the directions of arc shear force, arc pressure, and gravity all pointing towards the exit side of the pool at the keyhole rear side, a counterclockwise vortex is induced near the bottom of the weld pool. Therefore, in the rear wall of VPPA welding, particularly near the weld seam, two opposing vortices are observed. The change in the direction of gravity results in a difference in the accumulation zones of the two vortices. In vertical welding, oxides are uniformly distributed across the entire rear wall of the weld pool. However, in flat welding, oxides tend to accumulate more at the keyhole rear side near exit side.

Furthermore, there exists a Karman vortex street in the welding direction. By analyzing the cross-sectional view along the welding direction, Yan et al. discovered alternating occurrences of oxides within the weld seam [31]. Hence, it is evident that a Karman vortex street exists within the internal flow of the weld pool, which is particularly noticeable in the rear wall of the weld pool. Therefore, the theoretical model for the internal flow within the weld pool essentially encompasses two types of vortices. One type emerges as a Karman vortex street along the path from the front wall of the weld pool through the side wall to the rear wall, extending into the gradually solidifying zone. The axis of this vortex aligns parallel to the axis of the welding torch. The second type occurs along the direction from the entrance of the weld pool towards the exit, forming a vortex.

During the flat welding process, the notable expansion of flow channels exacerbates the internal vortex flow within the molten metal. Moreover, in the context of flat welding, there is a heightened propensity for the molten metal to gravitate towards the keyhole exit side. Consequently, this leads to a more substantial accumulation of metal and a higher temperature distribution at the keyhole exit side, thereby resulting in a reduced cooling rate for the molten metal within this region. In the course of flat welding, these intricate interplays among these factors cumulatively yield a conspicuous increase in grain size and the enlargement of coarse grain zones. The collective consequence of these intricate phenomena manifests as a marked deterioration in mechanical performance.

## 5. Conclusions

This study investigates the differences in the internal flow behavior of molten metal affected by gravity during VPPA welding, as well as the reasons for the resulting differences in temperature distribution and mechanical properties of the welded beads.

1. Within the weld pool, two types of vortex currents exist: one along the direction of welding and the other directed towards the base metal zone inside the weld pool. The presence of these vortex currents ensures thorough mixing of the molten metal within the pool, contributing to improved mechanical properties. This phenomenon is induced by the shear force, arc pressure, and gravity. Gravity can exacerbate the buildup of molten metal on the keyhole exit side.
2. Compared to the vertical position, flat position welding exhibits larger flow channels within the weld pool, especially along the sides of the keyhole. This results in more active internal vortex currents during flat welding. The increase in channel area during flat welding is attributed to heat accumulation.
3. During flat position, due to the influence of gravity, molten metal accumulates on one side of the keyhole exit, leading to elevated temperatures at the exit. This results in a larger zone with coarser grains on the exit side, consequently leading to inferior mechanical properties compared to the vertical welding position.

This research reveals critical characteristics of the internal flow behavior of molten metal at different positions during VPPA welding and demonstrates the significant impact of gravity on molten metal flow and material properties. These findings contribute to the optimization of VPPA welding processes, particularly in spatial locations, to enhance welding quality.

## Figures and Tables

**Table 1:** Chemical composition of base metal and feed wire (wt.%).

Material	Mg	Mn	Cr	Si	Fe	Zn	Cu	Al
5A06	5.80~6.80	0.50~0.80	-	≤0.40	≤0.40	≤0.20	≤0.10	Bla.
ER5183	4.50	0.70	0.15	≤0.40	≤0.40	≤0.25	≤0.10	Bla.
ER2319	0.02	0.20	-	0.20	≤0.40	≤0.10	5.80	Bla.

## Data Availability

Data is contained within the article.
